# Inclusion of patient-reported outcome instruments in US FDA medical device marketing authorizations

**DOI:** 10.1186/s41687-022-00444-z

**Published:** 2022-04-20

**Authors:** Sophia T. Matts, Christina M. Webber, Fraser D. Bocell, Brittany Caldwell, Allen L. Chen, Michelle E. Tarver

**Affiliations:** 1 Oak Ridge Institute for Science and Education (ORISE) Research Participation Program, Center for Devices and Radiological Health, U.S. Food and Drug Administration, Silver Spring, MD USA; 2 Center for Devices and Radiological Health, U.S. Food and Drug Administration, 10903 New Hampshire Ave, Silver Spring, MD 20993 USA

## Abstract

**Background:**

The U.S. Food and Drug Administration encourages the incorporation of the patient voice throughout the medical device total product lifecycle. This study examined the incorporation of patient-reported outcome (PRO) instruments in the evaluation of medical devices over a six-year period. PRO instruments used to inform study endpoints were extracted from the summary documents and clinical trial data of premarket authorizations posted on publicly available FDA databases between October 1, 2014—September 30, 2020.

**Results:**

PROs were included in 53% of authorizations, with 34% using PROs as primary and secondary endpoints. This study found that PRO instruments were used in each type of marketing authorization and in all medical specialties examined in this study.

**Conclusions:**

Expanding the current collaborative efforts to develop and modify PRO instruments may help to improve use of PROs in medical device evaluations.

## Background

Since the 2009 U.S. Food and Drug Administration (FDA) Guidance for Industry—Patient-Reported Outcome Measures: Use in Medical Product Development to Support Labeling Claims, there has been an emphasis on capturing the patient voice by incorporating patient-reported outcome (PRO) measures, also called PRO instruments, throughout the medical device total product lifecycle [1]. The FDA’s Center for Devices and Radiological Health (CDRH) explicitly expressed its commitment to partner with patients by incorporating the patient perspective in regulatory decisions, as detailed in the 2016–2017 Strategic Priorities [2]. The 21st Century Cures Act, passed in December 2016, encouraged the FDA to review and communicate patient experience data submitted in medical product reviews [3]. Additionally, as part of the Food and Drug Administration Reauthorization Act, the reauthorization of the Medical Device User Fee Amendments in 2017 (MDUFA IV) supported the appropriate use of PRO instruments in clinical investigations by improving regulatory predictability, maintaining least burdensome principles, and increasing transparency of patient input in medical device regulatory decision making [4]. All of these efforts have focused on improving the effective incorporation of PROs in the evaluation of medical products.

PRO instruments provide a way to directly measure the health condition of patients from their own report, without outside interpretation, and can be used to help evaluate the safety and effectiveness of a medical device [1]. PRO instruments can help inform the benefit-risk assessment of medical devices and are often included in the medical device labeling. To streamline the use of PRO instruments, CDRH has qualified tools through the Medical Device Development Tool (MDDT) program as scientifically acceptable measures of the patient experience within specific contexts of use. The MDDT program encourages collaboration on tool development which may potentially increase the number and use of qualified tools. As of October 1, 2020, four PRO instruments have been qualified through the program [5].

PROs can be used to inform primary, co-primary, or secondary endpoints in the trial [1]. While not all medical devices require a clinical trial for regulatory evaluation, many devices reviewed through Class III mechansims, De Novo or Humanitarian Use Devices (HUD) and some through class II mechaisms, are evaluated with clinical trials to support the regulatory decision. Medical device classification depends on the intended use and the indications for use of the device, as well as the device risk and the capability to appropriately mitigate the risks to health. Class I devices are subject to general controls and include those devices with the lowest risk [6, 7]. Class II devices are subject to general and special controls and may undergo regulatory review under a premarket notification [510(k)] demonstrating the device is “substantially equivalent” to a legally marketed device, known as a predicate device [6, 8, 9]. Novel devices of low to moderate risk lacking a predicate device may pursue the De Novo pathway that includes an evaluation of whether the information provides a reasonable assurance of safety and effectiveness and the risks can be appropriately mitigated with general, or general and special controls [10]. Class III devices are typically reviewed by the premarket approval (PMA) pathway. Class III devices are those that support or sustain human life, are of substantial importance in preventing impairment of human health, or which present a potential, unreasonable risk of illness or injury. Due to the level of risk associated with Class III devices, FDA has determined that general and special controls alone are insufficient to assure the safety and effectiveness of Class III devices [11]. A new indication for use or significant changes in the device design, performance, or the population in whom the device will be used for a class III device are submitted through a Panel-Track Supplement [12]. Lastly, Humanitarian Device Exemptions (HDEs) are granted for those devices intended to treat or diagnose patients with a rare disease or condition that affects less than 8,000 individuals annually. They are also exempt from effectiveness requirements [13, 14]. CDRH medical device review and monitoring throughout the total product lifecycle is organized by the Offices of Health Technology (OHT) 1–7, corresponding to different medical device product areas. (15; Table 1).

**Table 1:** Medical devices receiving marketing authorization fiscal year 2015 through fiscal year 2020 analyzed in this study, organized by CDRH OHT

Office of health technology	PMA	510 (k)	De Novo	HDE
OHT 1 (ophthalmic/anesthesia/respiratory/ear nose & throat/dental devices)	13%	23%	22%	8%
	n = 35	n = 47	n = 17	n = 1
OHT 2 (cardiovascular devices)	49%	21%	13%	25%
	n = 131	n = 42	n = 10	n = 3
OHT 3 (reproductive/gastro-renal/urological/general hospital devices and human factors)	7%	14%	23%	33%
	n = 18	n = 29	n = 18	n = 4
OHT 4 (neurological/psychiatric/physical medicine devices)	7%	20%	17%	0%
	n = 19	n = 40	n = 13	n = 0
OHT 5 (neurological/physical medicine devices)	7%	10%	18%	8%
	n = 20	n = 20	n = 14	n = 1
OHT 6 (orthopedic devices)	8%	5%	3%	25%
	n = 21	n = 11	n = 2	n = 3
OHT 7 (in vitro diagnostics*/radiological health)	9%	6%	5%	0%
	n = 24	n = 12	n = 4	n = 0
Total	n = 268	n = 201	n = 78	n = 12

Medical specialties within each OHT are listed in parentheses. Total number of marketing authorizations analyzed in this study, stratified by OHT and authorization type, are presented as “n” in each cell. *Only devices reviewed by the Clinical Chemistry advisory committee in OHT 7 were included in the study (numbers in table reflect this)

CDRH has made targeted efforts to encourage the appropriate incorporation of PROs across medical device areas. This study examined U.S. FDA marketing authorizations over a six-year period to better understand how and when PROs are included in medical device evaluation.

## Methods

### Cohort

Publicly available FDA documents with clinical trial data were reviewed to evaluate the use of PRO instruments in medical devices receiving marketing authorization from fiscal year 2015 through fiscal year 2020 (October 1, 2014 – September 30, 2020). Data was manually extracted from the following online FDA databases: Premarket Approval (PMA), 510(k), Device Classification Under Sect. 513(f) [2] (De Novo), and Humanitarian Device Exemption (HDE) [16–19]. Specifically, the documents reviewed included: Summary of Safety and Effectiveness Data (PMA Originals and Panel-Track Supplements), 510(k) Summaries, Decision Summaries (De Novos), and Summary of Safety and Probable Benefit (HDEs). Throughout this manuscript, these documents will be referred to collectively as summary documents.

Only authorized devices with summary documents posted publicly during the aforementioned interval were included, regardless of the date of the regulatory decision. Devices that were not authorized by the FDA were not included in this study. Duplicate summary documents were excluded from the analysis. Additionally, because many diagnostic devices are evaluated using clinical specimens or laboratory samples rather than human participants, all in vitro diagnostic devices, except those related to clinical chemistry in OHT 7 (e.g., continuous glucose monitors and insulin pumps), were excluded from the cohort [15].

### Variables of Interest

Each instance where a PRO instrument was used to inform study endpoints was extracted from the summary documents by two of the authors (S.T.M., C.M.W.). Data elements collected and recorded in a standard report form included: PRO instrument name, endpoint type (safety, effectiveness, both), endpoint positioning (primary, secondary, ancillary, not specified), device decision date, and device advisory committee [20].

### Analysis

Although many medical device marketing authorizations in this cohort were posted prior to the reorganization of CDRH in 2019, all devices analyzed in this study were stratified by OHT reflecting the 2019 reorganization. Devices were attributed to each OHT based on the associated advisory committees (Table 1). Quality checks between S.T.M. and C.M.W. to identify inconsistencies in data extraction were performed. When inconsistencies were identified, adjudication was performed by M.E.T., B.C., A.L.C., and F.D.B.

Data from the summary documents were analyzed using descriptive statistics in Tableau Desktop (Professional Edition, version 2020.2.1, Salesforce, Mountain View, CA) and Microsoft Excel for Mac (version 16.42, Microsoft, Redmond, WA).

## Results

### Description of the sample

Medical device marketing authorizations posted in public FDA databases fiscal year 2015 – fiscal year 2020 included 372 PMAs (Original and Panel Track), 302 510(k)s (with clinical trial data), 168 De Novos, and 15 HDEs. After removal of diagnostic devices and authorizations without summary data, the final population that was analyzed in this retrospective study included 268 PMAs, 201 510(k)s, 78 De Novos, and 12 HDEs (Table 1).

### PRO inclusion in summary documents

**Fig. 1:**
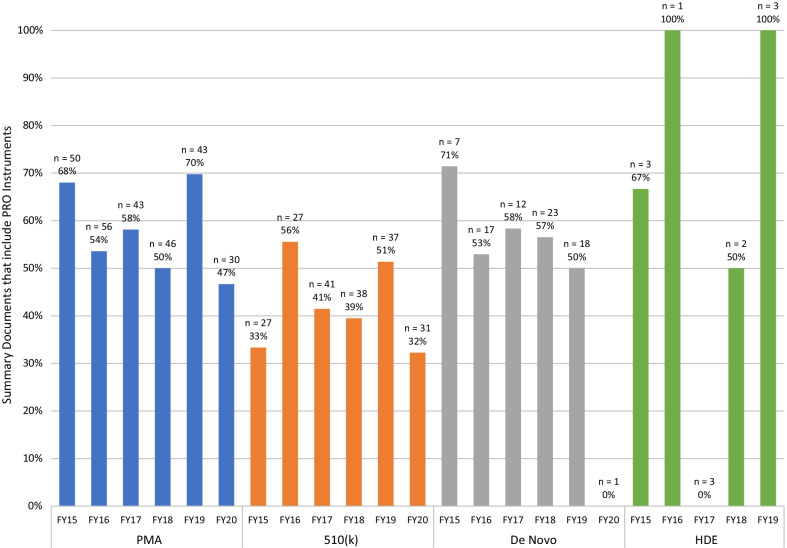
PRO inclusion in summary documents fiscal year 2015- fiscal year 2020. The 510(k) cohort consisted of cleared 510(k)s that included clinical trial data. The majority of De Novos granted in fiscal year 2020 did not have summary documents posted within the study window leading to the low *n* observed. There were no HDE approvals publicly posted in fiscal year 2020. FY = Fiscal year

PRO instruments were included in 52% of the summary documents analyzed in this study. Of the PMAs reviewed, 58% included PRO instruments. Original PMAs and Panel Track supplements included PRO instruments at similar rates, 57% and 59%, respectively. PROs were included in the summary documents of 42% of 510(k)s with clinical studies, 55% of De Novos, and 58% of HDEs, with some variation by fiscal year (Fig. 1).

### PRO Use by OHT

Across all marketing authorization types and in every medical device area examined in this study, PRO instruments were used. On average, there was greater than 80% inclusion of PROs in PMAs within OHTs 1, 3, 4, and 6, the offices evaluating ophthalmic; ear, nose, and throat; obstetrics and gynecology; urology; general surgical; and orthopedic devices, among other product areas (Table 2). On average, 64% of PMAs in OHT 5, evaluating evaluating neurological, psychiatric, and physical medicine devices, included a PRO instrument. OHT 2 (cardiovascular) experienced the lowest average inclusion of PROs among OHTs 1–6, with 42% inclusion in PMAs, and less in the other marketing authorization types. OHT 7, responsible for in vitro diagnostics and radiological health, had the lowest inclusion among all marketing authorization types, with 7% inclusion in PMAs.

**Table 2:** Percentage of PMA approvals that included PRO instruments by fiscal year

	OHT 1	OHT 2	OHT 3	OHT 4	OHT 5	OHT 6	OHT 7
Fiscal year 2015	100%	52%	100%	100%	67%	100%	0%
	n = 5	n = 23	n = 4	n = 6	n = 3	n = 5	n = 4
Fiscal year 2016	83%	30%	100%	100%	100%	100%	14%
	n = 6	n = 27	n = 4	n = 3	n = 4	n = 5	n = 7
Fiscal year 2017	100%	52%	100%	75%	50%	100%	0%
	n = 5	n = 25	n = 1	n = 4	n = 2	n = 2	n = 4
Fiscal year 2018	89%	29%	50%	50%	50%	100%	29%
	n = 9	n = 17	n = 2	n = 4	n = 4	n = 3	n = 7
Fiscal year 2019	100%	63%	80%		50%	100%	0%
	n = 5	n = 24	n = 5	n = 0	n = 4	n = 4	n = 1
Fiscal year 2020	40%	27%	100%	100%	67%	100%	0%
	n = 5	n = 15	n = 2	n = 2	n = 3	n = 2	n = 1
Fiscal year 2015-Fiscal year 2020 average	85%	42%	88%	85%	64%	100%	7%
	n = 35	n = 131	n = 18	n = 19	n = 20	n = 21	n = 24

### Endpoint positioning

**Fig. 2:**
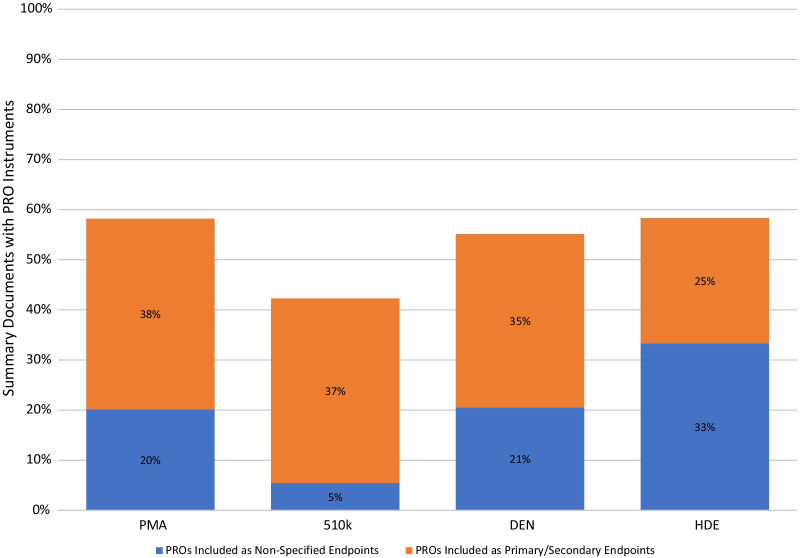
Inclusion of PRO in summary documents, separated by endpoint positioning. Summary documents were grouped by device authorization pathway (premarket approval (PMA), premarket notification [510(k)], Device Classification Under Sect. 510(f) [2] (De Novo), Humanitarian Device Exemption (HDE)). Percentage of marketing authorizations that included a PRO in the summary documents (total bar), separated by the marketing authorizations that used at least one PRO as a primary or secondary endpoint (orange) and those that included PROs as supporting data, either to support ancillary endpoints or without specifying endpoint positioning (blue)

To evaluate the specific usage of PROs, endpoint positioning (e.g., primary, secondary, ancillary) as listed in summary documents was analyzed. While an average of 53% of all marketing authorizations in this study included PRO instruments in their summary documents, only 34% included PROs as primary or secondary endpoints (Fig. 2). Twenty percent of marketing authorizations, on average, had summary documents that included PROs as supporting data, either to support ancillary endpoints or without specifying which endpoint the PRO supported.

### PRO instruments

Among the PRO instruments used in PMA approvals (original and panel track) examined in this study, disease-agnostic instruments like the Short Form Surveys (e.g. SF-36, SF-20, SF-12) and EuroQol-5D (EQ-5D) were the most prevalent, making up 10% and 8%, respectively. PRO instruments that are qualified MDDTs were also used in clinical trials designed to support PMAs and 510(k)s. Three qualified MDDTs, 1) Insulin Dosing Systems: Perceptions, Ideas, Reflections and Expectations (INSPIRE) Questionnaire, 2) Kansas City Cardiomyopathy Questionnaire (KCCQ), and 3) Minnesota Living with Heart Failure Questionnaire (MLHFQ), were all present in the dataset after their qualification [5]. The BREAST-Q and Patient-Reported Outcomes with LASIK (PROWL) questionnaires were excluded from analysis due to recent qualification in August 2020 and June 2021, respectively.

While general, disease-agnostic PRO instruments are consistently being used in submissions across medical device areas, some PRO instruments may have been independently developed for use in a single submission for marketing authorization even if an instrument already existed that could have been fit-for-purpose. Out of the 174 different instruments identified in PMA approvals, 112 (64%) were used in only one approval. These appeared in each medical product area, with the greatest utilization of these single-use PRO instruments in OHT 3 (71%; reproductive, gastro-renal, urological, general hospital devices, and human factors), OHT 4 (81%; surgical and infection control devices), and OHT 5 (73%; neurological and physical medicine devices) (data not shown).

## Discussion

Patient-centric evaluation of medical devices is a critical component of including the patient’s voice in the development of innovative medical devices. PRO instruments offer the opportunity to measure how a patient feels, functions, or survives based on a direct report from the patient, which can complement existing measures of safety and effectiveness. The use of PRO instruments throughout the healthcare ecosystem has been expanding from research into clinical practice, payor, and regulatory spaces [21–27]. This retrospective study examined the incorporation of the patient experience using PRO instruments in the evaluation of medical devices over a six-year period.

Consistent inclusion of PROs across the OHTs, apart from OHT 7, was found in the summary documents of PMAs, 510(k)s, De Novos, and HDEs analyzed in this study. On average, 52% of all summary documents for devices receiving marketing authorization from fiscal year 2015 through fiscal year 2020 contained at least one PRO instrument. However, others have analyzed PRO use in studies of medical devices and reported a lower percentage using PRO instruments. Vodicka et al. characterized all clinical trials registered on ClinicalTrials.gov between November 2007 and December 2013 and found that 30% of trials studying a medical device used a PRO instrument [28]. The current cohort only included clinical studies that resulted in marketing authorizations and analyzed a different timeframe, both of which may contribute to the differences in PRO inclusion rates between the two studies’ cohorts. Research has also been conducted to analyze PRO usage within drug approvals and labelling. A study examining drugs approved by the FDA between 2012 and 2016 with unique oncology indications reported a high PRO inclusion rate in the summary documents of 70% [29]. The increased rate could be a result of the study cohort focusing only on oncology drugs, an area where PROs can be particularly impactful due to the high burden of symptoms patients experience [24]. Similar analyses conducted on labeling claims of approved drugs in the United States reported PRO instrument inclusion rates of 30% and 24% for 1997–2002 and 2006–2010, respectively [30, 31]. Notable differences in the study cohorts exist between these studies and the current study cohort, namely the focus on PRO use in drug labeling claims rather than PRO inclusion in medical device summary documents. Overall, PRO instruments are being used in clinical trials and marketing authorizations across medical products to incorporate the patient’s experience.

Yearly variations in the number and types of devices submitted for marketing authorization partially account for the observed variations in the dataset. It is important to note, however, that a lack of PROs reported in a medical device area does not necessarily indicate that including the patient voice is not relevant in evaluation. It could indicate that further development of valid and reliable tools is needed to capture specific aspects of the patient experience in that area. Ongoing efforts by the FDA and international regulatory bodies encourage the incorporation of the patient experience across medical product areas, even those where the patient voice is traditionally not included [26, 27]. The FDA has issued guidance on the use of PROs to support labeling claims, as well as guidance on selecting, developing, modifying, and adapting PRO instruments to use in the evaluation of medical devices [1, 32]. Additionally, medical device developers can submit a PRO instrument for qualification in a specific context of use to CDRH’s MDDT program [5]. For example, the INSPIRE questionnaire was developed to assess the impact of automated insulin dosing systems on patients. This questionnaire was submitted to the MDDT program and qualified for that specific context of use [33]. Because the INSPIRE questionnaire can be used when evaluating clinical chemistry devices in OHT 7, it may increase the inclusion rate of PROs noted for this OHT. The qualification of PRO instruments may help facilitate greater incorporation of PROs in medical device clinical studies.

In this retrospective study, 64% of PRO instruments used were single-use PRO instruments. Instruments specific to a single manufacturer’s device evaluation program may add burden to manufacturers and patients participating in the development of multiple instruments. Consistent incorporation of fit-for-purpose PRO instruments within a given clinical research area may generate additional evidence to support its use by payors, healthcare providers, and regulators. By working collaboratively in the precompetitive space and potentially leveraging the MDDT qualification program, the burden of added costs and time could be distributed amongst manufacturers and greater efficiency could be realized.

While PROs in the majority of summary documents were specified as secondary endpoints, many PROs were included as supporting data to ancillary endpoints and were sometimes not specified as endpoints at all (Fig. 2). This suggests an opportunity for manufacturers and clinical trial developers to prespecify a priori how PROs are part of the investigational study. A priori defined endpoints may lead to more robustly collected PRO data and more informative analyses for regulatory and healthcare decisions. Prespecifying the endpoint positioning and definition can be useful as this helps clarify the research question related to the PRO and, thus, the intended interpretation of the PRO results [34].

The limitations of this retrospective study include manual extraction of data from summary documents where the PRO instruments were sometimes poorly defined or not described, making it challenging to identify whether and how a PRO instrument was used in the clinical study. Additionally, the data set was subject to the annual variation in medical device sponsors that seek and receive marketing authorization for their devices, which may have affected the year-to-year comparisons. However, a longitudinal look at this data is beneficial to examine trends and assess opportunities for future efforts.

In summary, this retrospective study presents longitudinal data on when PRO instruments have been used in medical device evaluation. Data were extracted directly from publicly accessible FDA databases containing the most recent medical devices receiving marketing authorizations. PRO instruments were included in summary documents, with inclusion rates varying by OHT. It is believed that precompetitive collaborations, as well as efforts to pursue MDDT qualification, could distribute the cost and time burden associated with new PRO instrument development. Further examination of other factors impacting the use of PRO instruments in medical device evaluation, including the parties and processes involved, may be warranted.

## Conclusion

PRO instruments can measure the patient’s perspective on their health condition and can be useful when evaluating medical devices. From fiscal year 2015 through fiscal year 2020, on average over half of all medical devices receiving marketing authorizations used at least one PRO instrument. Continued and expanded collaborative efforts have the potential to improve the consistency and efficiency surrounding the use of PRO instruments in medical device evaluations. Future research developing or modifying PRO instruments focused on areas where the patient’s perspective is not included in the development of new and novel medical devices may help to ensure outcomes important to patients are captured consistently.

## Data Availability

The datasets analyzed in the current study originated from the following databases: (1) Premarket Approval (PMA) [https://www.accessdata.fda.gov/scripts/cdrh/cfdocs/cfPMA/pma.cfm], (2) 510(k) [https://www.accessdata.fda.gov/scripts/cdrh/cfdocs/cfPMN/pmn.cfm], (3) Device Classification Under Sect. 513(f)(2) (De Novo) [https://www.accessdata.fda.gov/scripts/cdrh/cfdocs/cfPMN/denovo], and (4) Humanitarian Device Exemption (HDE) [https://www.accessdata.fda.gov/scripts/cdrh/cfdocs/cfHDE/hde.cfm].

## Abbreviations

PRO: Patient-reported outcome
FDA: U.S. food and drug administration
CDRH: Center for devices and radiological health
MDUFA IV: Medical device user fee amendments 2017
MDDT: Medical device development tool
HUD: Humanitarian use device
510(k): Premarket notification
PMA: Premarket approval
HDE: Humanitarian device extension
OHT: Office of health technology
FY: Fiscal year
EQ-5D: EuroQol-5D
INSPIRE: Insulin dosing systems, perceptions, ideas, reflections and expectations
KCCQ: Kansas city cardiomyopathy questionnaire
MLHFQ: Minnesota living with heart failure questionnaire
PROWL: Patient-reported outcomes with LASIK
